# Supplementary material for: The Small RNA Universe of Capitella teleta
Source: Front Mol Biosci. 2022 Feb 25;9:802814. doi: 10.3389/fmolb.2022.802814 (PMC8915122; doi:10.3389/fmolb.2022.802814)
Supplement: Supplementary file 1 [file DataSheet1.ZIP › Supplement/known/CAPTEscaffold_192_13675.pdf]

Provisional ID : CAPTEscaffold_192_13675
Score total : 220989.5
Score for star read(s) : 3.9
Score for read counts : 220979.2
Score for mfe : 1.8
Score for randfold : 1.6
Score for cons. seed : 3
Total read count : 433453
Mature read count : 432249
Loop read count : 8
Star read count : 1196

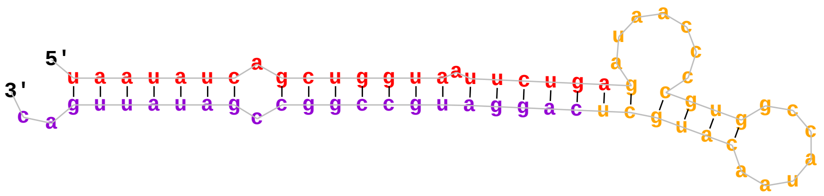

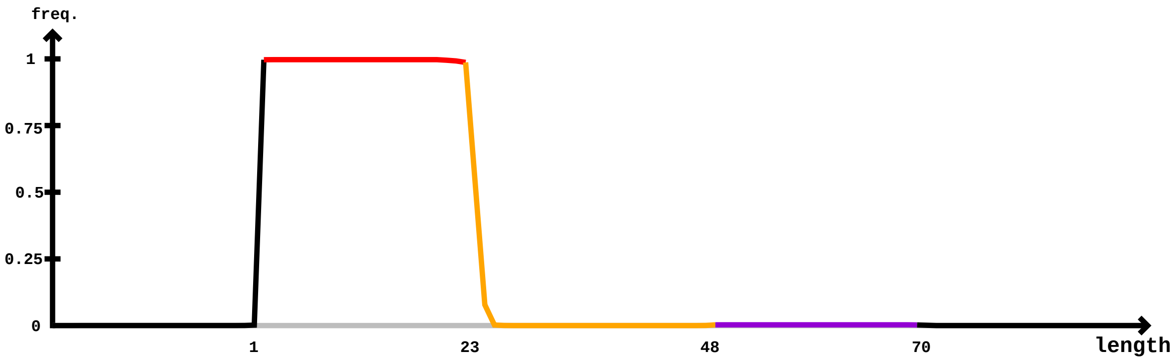


```
                        Mature                                          Star
5'- cuugcaugcguuugcuuggcuaauaucagcugguaauucugagauaaccccguggccauaacaugcucaggaugccggccgauauugaccaaucagacagcucuuugcuuau  -3'   obs
    cuugcaugcguuugcuuggcuaauaucagcugguaauucugagauaaccccguggccauaacaugcucaggaugccggccgauauugaccaaucagacagcucuuugcuuau        exp
    cuugcaugcguuugcuuggcuaauaucagcugguaauucugagauaaccccguggccauaacaugcucaggaugccggccgauauugaccaaucagacagcucuuugcuuau        known
    ...(((.(((((((.((((.(((((((.(((((((.(((((((.......((((.......)))))))))))))))))).))))))).)))).))))).))....)))....   reads   mm   sample

    cuugcaugcguuugcuuggc............................................................................................   1     0    seq
    .uugcaugcguuugcuuggc............................................................................................   41    0    seq
    .uugcaugcguuugcuuAgc............................................................................................   1     1    seq
    ..ugcaugcguuugcuuggc............................................................................................   5     0    seq
    ..................gcuaauaucagcugguaauucug.......................................................................   1     0    seq
    ..................gcuaauaucagcugguaauucuga......................................................................   80    0    seq
    ..................gcuaauauAagcugguaauucuga......................................................................   1     1    seq
    ..................gGuaauaucagcugguaauucuga......................................................................   2     1    seq
    ..................gcuaauaucagcugguaauucugU......................................................................   1     1    seq
    ..................Ucuaauaucagcugguaauucuga......................................................................   8     1    seq
    ..................Ucuaauaucagcugguaauucugag.....................................................................   1     1    seq
    ...................cuaauaucagcugguaauuc.........................................................................   1     0    seq
    ...................cuaauaucagcugguaauucu........................................................................   1     0    seq
    ...................cuaauaucagcugguaauucug.......................................................................   2     0    seq
    ...................cuaauaucGgcugguaauucuga......................................................................   2     1    seq
    ...................cuaUuaucagcugguaauucuga......................................................................   1     1    seq
    ...................cAaauaucagcugguaauucuga......................................................................   1     1    seq
    ...................cuaauaucagcugguaauuGuga......................................................................   1     1    seq
    ...................cCaauaucagcugguaauucuga......................................................................   1     1    seq
    ...................cuaauaucagcugguaauucugG......................................................................   1     1    seq
    ...................cuaauaucagcugguaauucuga......................................................................   549   0    seq
    ...................cuGauaucagcugguaauucuga......................................................................   1     1    seq
    ...................cuaauaucagcugguaauucugag.....................................................................   17    0    seq
    ...................Auaauaucagcugguaauucugag.....................................................................   1     1    seq
    ...................cuaauaucGgcugguaauucugag.....................................................................   5     1    seq
    ...................cuaauaucagcugguaauucugagU....................................................................   3     1    seq
    ...................cuaauaucagcugguaauucugaga....................................................................   2     0    seq
    ....................uaauaucagcugguaauu..........................................................................   10    0    seq
    ....................uaauauUagcugguaauuc.........................................................................   3     1    seq
    ....................uaGuaucagcugguaauuc.........................................................................   1     1    seq
    ....................uaauauAagcugguaauuc.........................................................................   3     1    seq
```

```
                    Mature                                       Star
cuugcaugcguuugcuuggcuaauaucagcugguaauucugagauaaccccguggccauaacaugcucaggaugccggccgauauugaccaaucagacagcucuuugcuuau
....................uaauaucagcugguaauuc.........................................................................  876   0  seq
....................uaauaucagcugguaaAuc.........................................................................  1     1  seq
....................uaauaucagcugUuaauuc.........................................................................  1     1  seq
....................uaauaucagcugguGauuc.........................................................................  1     1  seq
....................uaauGucagcugguaauuc.........................................................................  3     1  seq
....................uaauaucGgcugguaauuc.........................................................................  3     1  seq
....................uaauaucagcuAguaauuc.........................................................................  2     1  seq
....................Aaauaucagcugguaauuc.........................................................................  6     1  seq
....................Caauaucagcugguaauucu........................................................................  2     1  seq
....................uaauaucagcAgguaauucu........................................................................  1     1  seq
....................uaauaucagcugAuaauucu........................................................................  1     1  seq
....................uaauaucGgcugguaauucu........................................................................  92    1  seq
....................Aaauaucagcugguaauucu........................................................................  5     1  seq
....................uaaAaucagcugguaauucu........................................................................  1     1  seq
....................uaauaucaUcugguaauucu........................................................................  1     1  seq
....................uaauaucagcugguaauuAu........................................................................  1     1  seq
....................uaauaucagcugguGauucu........................................................................  1     1  seq
....................Naauaucagcugguaauucu........................................................................  2     1  seq
....................uaauaucagcugguaauucu........................................................................  1065  0  seq
....................uaauaAcagcugguaauucu........................................................................  1     1  seq
....................uaauGucagcugguaauucu........................................................................  4     1  seq
....................uaaAaucagcugguaauucug.......................................................................  2     1  seq
....................uaauaAcagcugguaauucug.......................................................................  2     1  seq
....................uaauaucagcugguGauucug.......................................................................  1     1  seq
....................uaauaucagcugguaaAucug.......................................................................  1     1  seq
....................Gaauaucagcugguaauucug.......................................................................  3     1  seq
....................uaauauAagcugguaauucug.......................................................................  3     1  seq
....................uaauaucagcugguaauucCg.......................................................................  1     1  seq
....................uaauaucagcugguaauucuC.......................................................................  1     1  seq
....................uaauauUagcugguaauucug.......................................................................  2     1  seq
....................uaauaucagcugguaauuGug.......................................................................  1     1  seq
....................uaauaucagcuAguaauucug.......................................................................  6     1  seq
....................uaauaucGgcugguaauucug.......................................................................  42    1  seq
....................Aaauaucagcugguaauucug.......................................................................  9     1  seq
....................uaauaucagcGgguaauucug.......................................................................  2     1  seq
....................uaauaucagcugguaauucuA.......................................................................  18    1  seq
....................uaauaucagcuggCaauucug.......................................................................  1     1  seq
....................uNaaucagcugguaauucug........................................................................  1     1  seq
....................uaauaucagcugguaauucAg.......................................................................  1     1  seq
....................uaauaucagcCgguaauucug.......................................................................  2     1  seq
....................uaauaucagcugguaauucug.......................................................................  2280  0  seq
....................uaauaucagcugguaauucuU.......................................................................  1     1  seq
....................uaauaucagAugguaauucug.......................................................................  3     1  seq
....................uaauaucagcugguaauucGg.......................................................................  2     1  seq
....................uaauaucagcugguaauuAug.......................................................................  1     1  seq
....................uaauaucagcugguaauAcug.......................................................................  2     1  seq
....................uaauaucagcugguaauucuCa......................................................................  21    1  seq
....................uaauaucagcuggAaauucuga......................................................................  72    1  seq
....................uUauaucagcugguaauucuga......................................................................  7     1  seq
....................uaauaucagcugguaauucCga......................................................................  51    1  seq
....................uaauaucagcuUguaauucuga......................................................................  24    1  seq
....................uaauaucUgcugguaauucuga......................................................................  12    1  seq
....................uCauaucagcugguaauucuga......................................................................  1     1  seq
....................uaauaucCgcugguaauucuga......................................................................  2     1  seq
....................uaauaucagcugguaauAcuga......................................................................  68    1  seq
....................uaauaucagcuAguaauucuga......................................................................  329   1  seq
....................uaauaAcagcugguaauucuga......................................................................  88    1  seq
....................uaauaucaCcugguaauucuga......................................................................  5     1  seq
....................uaauaucagcugguCauucuga......................................................................  1     1  seq
....................uaauauGagcugguaauucuga......................................................................  24    1  seq
....................uaauaucagcugguaauucugU......................................................................  248   1  seq
....................uaauaucagcGgguaauucuga......................................................................  22    1  seq
....................uaauCucagcugguaauucuga......................................................................  1     1  seq
....................uaauaucagcugguaauucugC......................................................................  4     1  seq
....................Gaauaucagcugguaauucuga......................................................................  142   1  seq
....................Caauaucagcugguaauucuga......................................................................  34    1  seq
....................Aaauaucagcugguaauucuga......................................................................  1099  1  seq
....................uaauaucagcugguaauuAuga......................................................................  117   1  seq
....................uaauaucagcugAuaauucuga......................................................................  35    1  seq
....................uaauaucagcugguaauuUuga......................................................................  25    1  seq
```

```
                    Mature                                              Star
cuugcaugcguuugcuuggcuaauaucagcugguaauucugagauaaccccguggccauaacaugcucaggaugccggccgauauugaccaaucagacagcucuuugcuuau
....................uaauaucagcugguaUuucuga......................................................................   31       1   seq
....................uaauaucagGugguaauucuga......................................................................   16       1   seq
....................uaauaucaUcugguaauucuga......................................................................   18       1   seq
....................uaNuaucagcugguaauucuga......................................................................   4        1   seq
....................uaauauUagcugguaauucuga......................................................................   162      1   seq
....................uaauaucagcugguaauucuga......................................................................   257278   0   seq
....................uaauaucagcugUuaauucuga......................................................................   24       1   seq
....................uaauaucagcuggGaauucuga......................................................................   12       1   seq
....................uaauaucagAugguaauucuga......................................................................   226      1   seq
....................uaauaucGgcugguaauucuga......................................................................   2187     1   seq
....................Naauaucagcugguaauucuga......................................................................   84       1   seq
....................uaUuaucagcugguaauucuga......................................................................   23       1   seq
....................uaauaGcagcugguaauucuga......................................................................   9        1   seq
....................uaauaucagcAgguaauucuga......................................................................   81       1   seq
....................uaauaucagcugguaauucuUa......................................................................   31       1   seq
....................uaauaucagcugguaauucAga......................................................................   61       1   seq
....................uaauaucagUugguaauucuga......................................................................   31       1   seq
....................uaauaucagcugguaauucGga......................................................................   28       1   seq
....................uaauaucagcuggCaauucuga......................................................................   37       1   seq
....................uaauaucagcugguaaAucuga......................................................................   55       1   seq
....................uaauaCcagcugguaauucuga......................................................................   29       1   seq
....................uaauaucagcugguaauucugG......................................................................   1609     1   seq
....................uaauaucagcugguaauuGuga......................................................................   20       1   seq
....................uaauaucagcugguaauGcuga......................................................................   5        1   seq
....................uaauaucagcugguaauCcuga......................................................................   31       1   seq
....................uaauaucagcuCguaauucuga......................................................................   13       1   seq
....................uaauUucagcugguaauucuga......................................................................   14       1   seq
....................uaaCaucagcugguaauucuga......................................................................   26       1   seq
....................uaauaucagcugguUauucuga......................................................................   12       1   seq
....................uaauaucagcugguaaGucuga......................................................................   15       1   seq
....................uaauaucagcugCuaauucuga......................................................................   21       1   seq
....................uaauaucagcCgguaauucuga......................................................................   52       1   seq
....................uaauaucagcugguaGuucuga......................................................................   30       1   seq
....................uaauaNcagcugguaauucuga......................................................................   1        1   seq
....................uaauaucagcugguaauucuAa......................................................................   141      1   seq
....................uaGuaucagcugguaauucuga......................................................................   51       1   seq
....................uNauaucagcugguaauucuga......................................................................   15       1   seq
....................uaaGaucagcugguaauucuga......................................................................   11       1   seq
....................uaauaucaAcugguaauucuga......................................................................   14       1   seq
....................uaauaucagcugguGauucuga......................................................................   81       1   seq
....................uaauaucagcugguaaCucuga......................................................................   21       1   seq
....................uaauGucagcugguaauucuga......................................................................   68       1   seq
....................uGauaucagcugguaauucuga......................................................................   50       1   seq
....................uaaAaucagcugguaauucuga......................................................................   167      1   seq
....................uaauauAagcugguaauucuga......................................................................   251      1   seq
....................uaauaucagcugguaauucCgag.....................................................................   22       1   seq
....................uUauaucagcugguaauucugag.....................................................................   2        1   seq
....................uaauaucCgcugguaauucugag.....................................................................   4        1   seq
....................uaauaucagcugguaauucuAag.....................................................................   117      1   seq
....................uaauaucaUcugguaauucugag.....................................................................   7        1   seq
....................uaauaucagcugguaauucAgag.....................................................................   27       1   seq
....................uaauaucagGugguaauucugag.....................................................................   6        1   seq
....................uaauaucagcugguaUuucugag.....................................................................   13       1   seq
....................uNauaucagcugguaauucugag.....................................................................   4        1   seq
....................Caauaucagcugguaauucugag.....................................................................   22       1   seq
....................uaauaucagcuUguaauucugag.....................................................................   9        1   seq
....................uaauaucagcugguaauucGgag.....................................................................   13       1   seq
....................uaauGucagcugguaauucugag.....................................................................   27       1   seq
....................uaauaucagcuggCaauucugag.....................................................................   22       1   seq
....................uaauaucagcugguaauucugCg.....................................................................   1        1   seq
....................uaauaucagUugguaauucugag.....................................................................   16       1   seq
....................uaauaucagcugguaauAcugag.....................................................................   40       1   seq
....................uaauauGagcugguaauucugag.....................................................................   15       1   seq
....................uaauaucagcugguaauucuCag.....................................................................   9        1   seq
....................uaauaucagcugguaauucugUg.....................................................................   11       1   seq
....................uaauaucagcugguaauuAugag.....................................................................   43       1   seq
....................uaaAaucagcugguaauucugag.....................................................................   80       1   seq
....................uaUuaucagcugguaauucugag.....................................................................   10       1   seq
....................uaauauAagcugguaauucugag.....................................................................   86       1   seq
....................uaauaGcagcugguaauucugag.....................................................................   1        1   seq
```

```
                    Mature                          Star
cuugcaugcguuugcuuggcuaauaucagcugguaauucugagauaaccccguggccauaacaugcucaggaugccggccgauauugaccaaucagacagcucuuugcuuau
....................uaauaucagcugguaGuucugag.....................................................................     7       1    seq
....................uaauaucagcugguaaGucugag.....................................................................     6       1    seq
....................uaauaucaAcugguaauucugag.....................................................................     10      1    seq
....................Naauaucagcugguaauucugag.....................................................................     41      1    seq
....................uaauaucagcugguaauucugaU.....................................................................     143     1    seq
....................uaauaucagcugUuaauucugag.....................................................................     10      1    seq
....................uaaGaucagcugguaauucugag.....................................................................     9       1    seq
....................uaauaCcagcugguaauucugag.....................................................................     11      1    seq
....................Gaauaucagcugguaauucugag.....................................................................     50      1    seq
....................uaauaucagcugguaauucugaA.....................................................................     214     1    seq
....................uaGuaucagcugguaauucugag.....................................................................     16      1    seq
....................uaauaucagAugguaauucugag.....................................................................     114     1    seq
....................uaauaucagcugguaauucugaC.....................................................................     16      1    seq
....................uaauaucagcugguaauuUugag.....................................................................     6       1    seq
....................uaauaucGgcugguaauucugag.....................................................................     22248   1    seq
....................uaauaucagcugguaauucugGg.....................................................................     21      1    seq
....................uaauaucagcuggGaauucugag.....................................................................     5       1    seq
....................uaauaucagcugAuaauucugag.....................................................................     14      1    seq
....................uaauaucagcAgguaauucugag.....................................................................     29      1    seq
....................uaauaucUgcugguaauucugag.....................................................................     7       1    seq
....................uaauaucagcCgguaauucugag.....................................................................     14      1    seq
....................uaauaucagcugguUauucugag.....................................................................     9       1    seq
....................uaauaucagcugguaauucugag.....................................................................     103161  0    seq
....................uaauaucagcugguaaAucugag.....................................................................     25      1    seq
....................uaauaucagcGgguaauucugag.....................................................................     9       1    seq
....................uaNuaucagcugguaauucugag.....................................................................     1       1    seq
....................uaauaucagcuAguaauucugag.....................................................................     157     1    seq
....................uaauaucagcuggAaauucugag.....................................................................     24      1    seq
....................uaaCaucagcugguaauucugag.....................................................................     20      1    seq
....................Aaauaucagcugguaauucugag.....................................................................     417     1    seq
....................uaauaucagcugguGauucugag.....................................................................     34      1    seq
....................uaauaucagcugguaauGcugag.....................................................................     3       1    seq
....................uaauaucagcugCuaauucugag.....................................................................     5       1    seq
....................uGauaucagcugguaauucugag.....................................................................     20      1    seq
....................uaauaucagcugguaauuGugag.....................................................................     6       1    seq
....................uaauaucagcugguaaCucugag.....................................................................     8       1    seq
....................uaauaucaCcugguaauucugag.....................................................................     2       1    seq
....................uaauUucagcugguaauucugag.....................................................................     10      1    seq
....................uaauaAcagcugguaauucugag.....................................................................     33      1    seq
....................uaauaucagcuCguaauucugag.....................................................................     8       1    seq
....................uaauaucagcugguaauCcugag.....................................................................     14      1    seq
....................uaauauUagcugguaauucugag.....................................................................     55      1    seq
....................uaauaucagcugguaauucuUag.....................................................................     6       1    seq
....................uaauaucagcAgguaauucugaga....................................................................     7       1    seq
....................uaGuaucagcugguaauucugaga....................................................................     5       1    seq
....................uaauaucagcugguaauucugagC....................................................................     117     1    seq
....................uaauUucagcugguaauucugaga....................................................................     1       1    seq
....................uaauaucagcugCuaauucugaga....................................................................     1       1    seq
....................uaauauUagcugguaauucugaga....................................................................     13      1    seq
....................uaauaucagcugguaauucugCga....................................................................     1       1    seq
....................uaauauGagcugguaauucugaga....................................................................     2       1    seq
....................uaauaucagUugguaauucugaga....................................................................     2       1    seq
....................uaauaucagcugguaauuUugaga....................................................................     2       1    seq
....................uaauaucCgcugguaauucugaga....................................................................     1       1    seq
....................uaauaucagcugguaauucCgaga....................................................................     4       1    seq
....................uaauaucagcuAguaauucugaga....................................................................     25      1    seq
....................uaauaucagcCgguaauucugaga....................................................................     9       1    seq
....................uaauaucagcugguaauucuAaga....................................................................     25      1    seq
....................uaauaucaUcugguaauucugaga....................................................................     2       1    seq
....................uaauaucagGugguaauucugaga....................................................................     2       1    seq
....................uaauaGcagcugguaauucugaga....................................................................     1       1    seq
....................uaauaucagcugguaaCucugaga....................................................................     3       1    seq
....................uaauaucagcugguaauucGgaga....................................................................     2       1    seq
....................uaauaucagcugguaauuGugaga....................................................................     1       1    seq
....................uaauaucagcuCguaauucugaga....................................................................     3       1    seq
....................uaauaucagcugguaauucugagN....................................................................     1       1    seq
....................uaauaucagcugguaauucugaUa....................................................................     8       1    seq
....................uaauaucagAugguaauucugaga....................................................................     34      1    seq
....................uaUuaucagcugguaauucugaga....................................................................     1       1    seq
....................uaauaucagcugguaUuucugaga....................................................................     4       1    seq
```

```
                    Mature                                          Star
cuugcaugcguuugcuuggcuaauaucagcugguaauucugagauaaccccguggccauaacaugcucaggaugccggccgauauugaccaaucagacagcucuuugcuuau
....................uaauaucagcugguaauAcugaga....................................................................   6      1   seq
....................uaauaucGgcugguaauucugaga....................................................................   3404   1   seq
....................uaauaucagcuUguaauucugaga....................................................................   1      1   seq
....................Aaauaucagcugguaauucugaga....................................................................   106    1   seq
....................uaauaucagcugguaauucugGga....................................................................   2      1   seq
....................uaauauAagcugguaauucugaga....................................................................   23     1   seq
....................uaauaucagcugguaauucugUga....................................................................   2      1   seq
....................uaauaucagcugguaauucugaAa....................................................................   50     1   seq
....................Caauaucagcugguaauucugaga....................................................................   4      1   seq
....................uaauaucagcugguaauucugagU....................................................................   4003   1   seq
....................uaaGaucagcugguaauucugaga....................................................................   1      1   seq
....................Gaauaucagcugguaauucugaga....................................................................   14     1   seq
....................uaauaucagcugguaauucAgaga....................................................................   8      1   seq
....................uaauGucagcugguaauucugaga....................................................................   2      1   seq
....................uaauaucagcugguGauucugaga....................................................................   12     1   seq
....................uaauaucagcugUuaauucugaga....................................................................   3      1   seq
....................uaauaucagcugguaauuAugaga....................................................................   14     1   seq
....................uaauaucagcugAuaauucugaga....................................................................   7      1   seq
....................uaaCaucagcugguaauucugaga....................................................................   4      1   seq
....................uaauaucaAcugguaauucugaga....................................................................   4      1   seq
....................uaauaucagcuggCaauucugaga....................................................................   2      1   seq
....................uaauaucagcGgguaauucugaga....................................................................   3      1   seq
....................uaauaucagcugguaauucugagG....................................................................   77     1   seq
....................uaauaucagcugguaaAucugaga....................................................................   6      1   seq
....................uaauaucagcugguaauCcugaga....................................................................   2      1   seq
....................uaauaucagcugguaauucuUaga....................................................................   4      1   seq
....................uaauaAcagcugguaauucugaga....................................................................   5      1   seq
....................uaauaucagcugguaauucugaCa....................................................................   4      1   seq
....................uGauaucagcugguaauucugaga....................................................................   5      1   seq
....................uaauaucagcuggAaauucugaga....................................................................   10     1   seq
....................uNauaucagcugguaauucugaga....................................................................   1      1   seq
....................Naauaucagcugguaauucugaga....................................................................   12     1   seq
....................uaaAaucagcugguaauucugaga....................................................................   12     1   seq
....................uaauaucagcugguaauucugaga....................................................................   24757  0   seq
....................uaauaucagcugguaauucugagaA...................................................................   632    1   seq
....................uaauaucGgcugguaauucugagau...................................................................   1      1   seq
....................uaauaucagcugguaauucugagaG...................................................................   4      1   seq
....................Aaauaucagcugguaauucugagau...................................................................   1      1   seq
....................uaauaucagcugguaauucugagUu...................................................................   21     1   seq
....................uaauaucagcugguaauucugagau...................................................................   118    0   seq
....................uaauaucagcugguaauucugagauU..................................................................   81     1   seq
....................uaauaucagcugguaauucugagUua..................................................................   3      1   seq
....................uaauaucagcugguaauucugagaCa..................................................................   1      1   seq
....................uaauaucagcugguaauucugagaAa..................................................................   33     1   seq
....................uaauaucagcugguaauucugagaua..................................................................   4      0   seq
....................uaauaucagcugguaauucugagauaU.................................................................   1      1   seq
....................uaauaucagcugguaauucugagaAaa.................................................................   9      1   seq
....................uaauaucagcugguaauucugagauaac................................................................   1      0   seq
.....................aauaucagcugguaauucug.......................................................................   1      0   seq
.....................Uauaucagcugguaauucuga......................................................................   17     1   seq
.....................aauaucagcugguaauucuga......................................................................   19     0   seq
.....................aauaucagcugguaauucugG......................................................................   1      1   seq
.....................aauaucGgcugguaauucugag.....................................................................   3      1   seq
.....................aauaucagcugguaauucugag.....................................................................   10     0   seq
.....................Uauaucagcugguaauucugag.....................................................................   6      1   seq
.....................aauaucagcugguaauucugaga....................................................................   30     0   seq
.....................Uauaucagcugguaauucugaga....................................................................   2      1   seq
.....................aauaucGgcugguaauucugaga....................................................................   24     1   seq
......................auaucagcugguaauucuga......................................................................   3      0   seq
......................auaucagcugguaauucugag.....................................................................   1      0   seq
......................auaucagcugguaauucugaga....................................................................   2      0   seq
.......................uaucagcugguaauucuga......................................................................   5      0   seq
.......................uaucagcugguaauucugaga....................................................................   2      0   seq
........................aucagcugguaauucuga......................................................................   3      0   seq
........................aucagcugguaauucugag.....................................................................   1      0   seq
..........................cagcugguaauucugaga....................................................................   2      0   seq
..........................................gauaaccccguggccauaacaugcuc............................................   1      0   seq
...........................................auaaccccguggccauaacaugcu.............................................   4      0   seq
............................................uaaccccguggccauaacaugc..............................................   2      0   seq
............................................uaaccccguggccauaacaugcu.............................................   1      0   seq
```

Mature **Star**

```
cuugcaugcguuugcuuggcuaauaucagcugguaauucugagauaaccccguggccauaacaugcucaggaugccggccgauauugaccaaucagacagcucuuugcuuau
.................................................ccguggccauaacaugcucaggaugcc....................................    1     0    seq
..................................................................ucaggaugccggccgauau...........................    1     0    seq
..................................................................ucaggaugccggccgauauug.........................    1     0    seq
..................................................................ucaggaugccggAcgauauuga........................    1     1    seq
..................................................................ucaggaugccggccAauauuga........................    1     1    seq
..................................................................ucaggaugccggccgauauuga........................    165   0    seq
..................................................................ucaggaugccggccgauauugac.......................    16    0    seq
...................................................................caggaugccggccgauau...........................    1     0    seq
...................................................................caggaugccggccgauauuga........................    24    0    seq
...................................................................caggaugccggUcgauauugac.......................    1     1    seq
...................................................................caggaugcGggccgauauugac.......................    1     1    seq
...................................................................caggaugccggccgauauugac.......................    934   0    seq
...................................................................caggaugccggAcgauauugac.......................    1     1    seq
...................................................................caggaugccggccgaAauugac.......................    1     1    seq
...................................................................caggaugcUggccgauauugac.......................    1     1    seq
...................................................................caggaAgccggccgauauugac.......................    1     1    seq
...................................................................caggaugccggcAgauauugac.......................    1     1    seq
...................................................................caggaugccggccAauauugac.......................    1     1    seq
...................................................................caggaugccggccgauauugaG.......................    1     1    seq
...................................................................caggauAccggccgauauugac.......................    1     1    seq
...................................................................caggaugccggcUgauauugac.......................    3     1    seq
...................................................................caggaugccggccgauauugacA......................    7     1    seq
...................................................................caggaugccggccgauauugacU......................    14    1    seq
...................................................................caggaugccggccgauauugacc......................    1     0    seq
....................................................................aggaugccggccgauauuAac.......................    1     1    seq
....................................................................aggaugccggccgauauugac.......................    11    0    seq
....................................................................aggaugccggccgauauugacA......................    2     1    seq
....................................................................aggaugccggccgauauugacU......................    3     1    seq
.........................................................................................caaucagacagcucuuug.....    1     0    seq
.........................................................................................caaucagacagcucuuugc....    2     0    seq
```
